# Supplementary material for: Content-rich biological network constructed by mining PubMed abstracts
Source: BMC Bioinformatics. 2004 Oct 8;5:147. doi: 10.1186/1471-2105-5-147 (PMC528731; doi:10.1186/1471-2105-5-147)
Supplement: Additional File 2 — The original results of the above study (non-essential files are deleted to keep the file size under the limit set by BMC bioinformatics). [file 1471-2105-5-147-S2.bz2 › chilibotAdditionalFile2/dip10/515ID8799122E10818/html/ESR1_CREBBP.html]

 


 **ESR1** and **CREBBP** 
  
Found 57 abstracts in PubMed, retrieved 10.  
 

 What does Google say? 
 PDF only 
| .edu only 

---

**Interactive relationship** (e.g. stimulation, inhibition, etc)

**Neutral relationship**- RTS  [ **CREBBP** ]  offers new promising possibilities in the postgenomic era  [ **ESR1** ] .  Ref: 12570786 Curr Protein Pept Sci, 2003
- Ligand Independent Interactions of p160 SRC Coactivators and CBP  [ **CREBBP** ]  with Estrogen Receptor alpha  [ **ESR1** ]  Regulation by Phosphorylation Sites in the A B Region Depends on Other Receptor Domains.  Ref: 12714702 Mol Endocrinol< MedlineTA>Mol Endocrinol, 2003
- Mediator and p300 CBP  [ **CREBBP** ]  steroid receptor coactivator complexes have distinct roles, but function synergistically, during estrogen receptor alpha  [ **ESR1** ]  dependent transcription with chromatin templates.  Ref: 12482985 Mol Cell Biol, 2003
- These results suggest that 17beta estradiol bound estrogen receptor  [ **ESR1** ]  may inhibit nuclear factor kappaB dependent transcription of RANTES gene by competing with p65 for limiting amounts of CREB binding protein  [ **CREBBP** ] .  Ref: 12603855 J Invest Dermatol, 2003
- Further, 4EM mediated transcription in ER alpha  [ **ESR1** ] , like estrogen, was enhanced in the presence of coactivators, steroid receptor coactivator 1 SRC 1, CREB binding  [ **CREBBP** ]  proteins CBP  [ **CREBBP** ] , and E6 associated protein E6 AP.  Ref: 12732288 J Steroid Biochem Mol Biol, 2003

**Non-interactive relationship** (e.g. studied together, co-existance, homology, etc.)

- We have used a biochemical approach, including an in vitro chromatin assembly and transcription system, to examine the functional role for Mediator in the transcriptional activity of estrogen receptor alpha  [ **ESR1** ]  ERalpha with chromatin templates, as well as functional interplay between Mediator and p300 CBP  [ **CREBBP** ]  during ERalpha dependent transcription.  Ref: 12482985 Mol Cell Biol, 2003
